# Hyperglycemia‐Enhanced Neutrophil Extracellular Traps Drive Mucosal Immunopathology at the Oral Barrier

**DOI:** 10.1002/advs.202407346

**Published:** 2024-11-05

**Authors:** Qian Wang, Weimin Lin, Kexin Lei, Hui Wang, Xiaohan Zhang, Shuang Jiang, Danting Zhang, Wen Wang, Shuqin Cao, Yuyu Li, Bo Yu, Yuan Wang, Qi Yin, Quan Yuan

**Affiliations:** ^1^ State Key Laboratory of Oral Diseases & National Center for Stomatology & National Clinical Research Center for Oral Diseases West China Hospital of Stomatology Sichuan University Chengdu Sichuan 610041 China; ^2^ Department of Prosthodontics Sichuan Provincial People's Hospital University of Electronic Science and Technology of China Chengdu 611731 China; ^3^ Department of Basic and Translational Sciences Laboratory of Innate Immunity and Inflammation Penn Dental Medicine University of Pennsylvania Philadelphia PA 19104 USA; ^4^ Hebei Key Laboratory of Stomatology Hebei Clinical Research Center for Oral Diseases Hebei Medical University Shijiazhuang Hebei 050017 China; ^5^ Division of Preventive and Restorative Sciences School of Dentistry University of California Los Angeles Los Angeles CA 90095 USA; ^6^ Department of Implantology West China Hospital of Stomatology Sichuan University Chengdu Sichuan 610041 China

**Keywords:** glucose transporter 1, glycolysis, hyperglycemia, neutrophils, oral mucosal immunity, type 2 diabetes

## Abstract

Type 2 diabetes (T2D) is a risk factor for mucosal homeostasis and enhances the susceptibility to inflammation, in which neutrophils have been increasingly appreciated for their role. Here, barrier disruption and inflammation are observed at oral mucosa (gingiva) of T2D patients and mice. It is demonstrated that neutrophils infiltrate the gingival mucosa of T2D mice and expel obvious neutrophil extracellular traps (NETs), while removal of NETs alleviates the disruption of mucosal barrier. Mechanistically, gingival neutrophils released NETs are dependent of their metabolic reprogramming. Under hyperglycemic condition, neutrophils elevate both glucose incorporation and glycolysis via increased expression of GLUT1. Moreover, significantly increased levels of NETs are observed in local gingival lesions of patients, which are associated with clinical disease severity. This work elucidates a causative link between hyperglycemia and oral mucosal immunopathology, mediated by the altered immuno‐metabolic axis in neutrophil, thereby suggesting a potential therapeutic strategy.

## Graphical abstract

Hyperglycemia contributes to mucosal immunopathology in several barrier tissues, as seen with barrier destruction at the oral mucosa (gingiva); which promotes periodontitis via induction of neutrophil extracellular traps (NETs) after reprogramming neutrophil metabolism, constituting an immuno‐metabolic mechanism linking oral and systemic health.

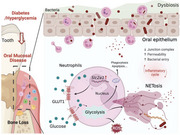

## 1 Introduction

Type 2 diabetes (T2D) is a complex metabolic disorder characterized by chronic hyperglycemia. The incidence of T2D‐associated infections is increasing globally,^[^ ^1^ ^]^ with a growing evidence suggests that hyperglycemia compromises the barrier integrity and disrupts mucosal homeostasis.^[^ ^2^, ^3^ ^]^ Oral mucosa, especially gingival site, is more inclined to inflame in diabetic individuals.^[^ ^4^, ^5^, ^6^ ^]^ Inflammatory destruction of gingival mucosa along with alveolar bone surrounding the tooth has been found to be both more prevalent and severe in patients with T2D.^[^ ^7^, ^8^ ^]^ Early studies thus far have reported that hyperglycemia promotes the susceptibility of gingival mucosa to infection and persistent inflammation.^[^ ^9^ ^]^

Gingival mucosa forms a physical, chemical, and immunological barrier against invading microbes. Mounting evidences demonstrate destruction of gingival mucosal epithelium as a pivotal pathological basis for periodontitis.^[^ ^10^, ^11^ ^]^ Studies performed on the gut, skin, and esophagus have demonstrated that inflammatory responses can be induced as a consequence of an opening of the epithelial barrier, leading to a vicious cycle where the subepithelial inflammation itself continues to maintain damaged and open barriers.^[^ ^12^ ^]^ Hyperglycemia has been long recognized to affect epithelial barrier functions and thus bacterial invasion of the gingiva.^[^ ^13^ ^]^ In this condition, dysregulated mucosal inflammation triggers destruction of soft tissues and supporting alveolar bone, leading to tooth loss in severe cases. Yet, the detailed pathogenic mechanism underlying impairment of oral mucosal barrier in hyperglycemia remains poorly understood.

Neutrophil is a sentinel cell that assists mucosal epithelium to maintain the integrity of immunologic barrier,^[^ ^14^ ^]^ and their functions are essential for regulation of mucosal homeostasis.^[^ ^15^, ^16^ ^]^ Increased neutrophil infiltration and activation are associated with oral mucosal inflammation,^[^ ^17^, ^18^ ^]^ and neutrophil hyperactivation has been also documented in gingival samples of T2D patients.^[^ ^19^ ^]^ Neutrophil extracellular traps (NETs) released (termed NETosis) by activated neutrophils following pathogens’ stimulation are instrumental to protection against invasive infections. Through the process of NETosis, intracellular autoantigens and enzymes are released which would be detrimental to surrounding tissues. Recently, NETs have been proposed as dominant drivers in immunopathology of several chronic diseases impacted by metabolic disorders. NET structures and NET‐associated proteins have been documented both in tissue lesions and in circulation of patients with diabetes.^[^ ^20^, ^21^ ^]^ Hyperglycemia has been also shown to directly mediate NETs.^[^ ^22^ ^]^ Notably, a new work provided evidence for the role of NETs as early disease triggers of pathogenic inflammation in chronic periodontitis.^[^ ^23^ ^]^ These links raise a question of whether NETs are involved in gingival epithelial injury and contribute to periodontitis progression in T2D. Furthermore, the mechanism by which hyperglycemia mediates NETs formation or impairs NETs clearance in oral mucosal lesions is not known.

In this work, we use a diabetic mouse model to study the oral mucosal immunopathology developed in patients with T2D, and which is associated with NETs accumulation in gingiva. We aim to address the functional role of NETs in the induction of oral mucosal inflammation and disruption of barrier integrity. Moreover, the potential mechanisms by which hyperglycemia promotes NETs are further explored.

## 2 Results

### 2.1 Diabetes Induces an Immunopathology at Oral Mucosal Barrier

**Figure 1:**
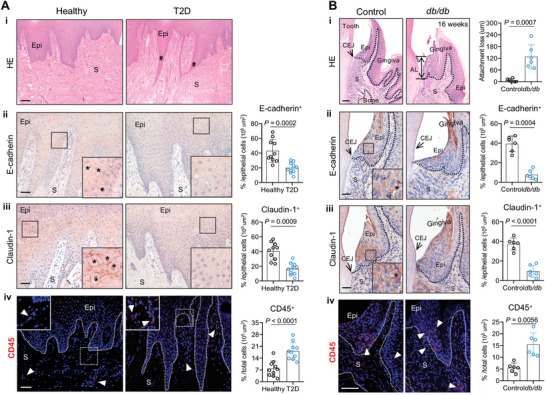
Oral mucosal immunopathology in diabetic patients and mice. (A) Gingival biopsies from patients. (A‐i) Hematoxylin and eosin (H&E) staining of gingival tissue sections (black hashes indicate gingival epithelial spikes). Epi, epithelium; S, stroma. Scale bar: 100 µm. (A‐ii, iii) Immunohistochemical (IHC) staining of E‐cadherin and Claudin‐1 in gingival biopsies (black asterisks depict positive cells). Right: graphs showing the percentage of positive E‐cadherin and Claudin‐1 cells in epithelium. Scale bars, 50 µm. (A‐iv) Immunofluorescence (IF) staining for CD45 in gingival lesions. And white triangles indicate CD45^+^ leukocytes. Right: quantification of the mean fluorescence intensity (MFI) of CD45 staining (*n* = 10, one‐way ANOVA). Scale bar: 50 µm. (B) Gingival samples from animal models. *db/db* group: *db/db* mice, Control group: heterozygote mice. (B‐i) H&E staining of gingival tissue sections (black arrowhead depicts CEJ and black lines indicate mucosal attachment loss). Right: quantification of mucosal attachment loss (*n* = 6, two‐tail Student *t*‐test). AL, attachment loss; CEJ, cementoenamel junction; Epi, epithelium; S, stroma. Scale bar: 50 µm. (B‐ii, iii) IHC staining of E‐cadherin and Claudin‐1 in gingival tissues (black asterisks depict positive cells) from control and *db/db* mice. Right: graphs showing the percentage of positive E‐cadherin and Claudin‐1 cells in epithelium (*n* = 6, one‐way ANOVA). Scale bars, 25 µm. (B‐iv) IF staining for CD45 in mouse gingival tissue sections (white triangles indicate CD45^+^ leukocytes). Right: graphs showing the MFI of CD45 staining (*n* = 6, one‐way ANOVA). Scale bar: 10 µm. All data are presented as mean ± SD.

For this study, we first assembled a cohort of patients with T2D in the absence of any known confounders and healthy control (Table S1, Supporting Information). Histological staining of the gingival samples from T2D patients showed a slight thickening of keratinized epithelium, epithelial spikes extended, and subepithelial dense collagen fibers arranged in bundles of courses (**Figure** **1**A‐i). Compared with healthy control, gingival mucosa of patients had few positive immunostainings of E‐cadherin and Claudin‐1 (a well‐established marker of tight and adherent junctions) between adjacent epithelial cells (Figure 1A‐ii,iii). As visualized by immunostaining, there was more prominent CD45^+^ immune cell infiltration among epithelial spikes in T2D, though still localized infiltration in healthy gingiva (Figure 1A‐iv).

We further carried out observations in a type 2 diabetic mouse model that develops hyperglycemia spontaneously.^[^ ^24^ ^]^ The blood glucose and body weight were measured at different ages, both of them were significantly increased after 8 weeks old (Figure S1A,B, Supporting Information). Notably, *db/db* mice spontaneously developed periodontal bone loss by 8 weeks of age, and this loss became more severe at 16 weeks of age (Figure S1C, Supporting Information). Gingival tissues from *db/db* mice presented attachment loss at junctional epithelium supporting the dentition (Figure 1B‐i). Consistent with results from T2D patients, expression of either E‐cadherin or Claudin‐1 at epithelial barrier, indicated not well‐developed intercellular junction architectures in *db/db* gingiva (Figure 1B‐ii,iii). In addition, gingival mucosa of *db/db* mice induced more infiltration of CD45^+^ immune cells near the cervical area compared with control mice (Figure 1B‐iv).

To confirm that spontaneous periodontitis in *db/db* mice was associated with the developed diabetic conditions, we used another mouse model with high‐fat diet/streptozotocin (STZ)‐induced characteristic hyperglycemia (Figure S2A–C, Supporting Information). With the induction of hyperglycemia, STZ‐induced T2D mice showed obvious periodontal bone loss, attachment loss and aggravated histopathology of gingival mucosa compared with control (Figure S2D,E, Supporting Information). Overall, these results reflect an imbalance of oral mucosal homeostasis in setting of T2D.

### 2.2 NETosis is Evident in Oral Mucosal Immunopathology of Diabetic Mice

**Figure 2:**
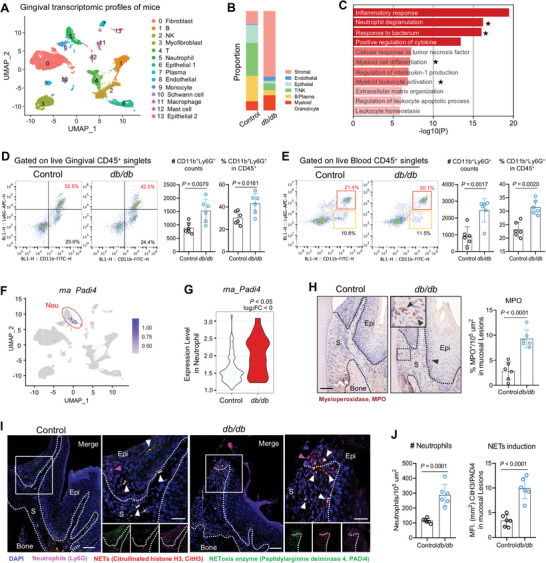
NETosis is evident in oral mucosal lesions of *db/db* mice. (A) Single‐cell RNA sequencing (scRNA‐seq) data about gingival mucosal map of control and *db/db* mice. UMAP plot representation of identified cell types (a total of 14 clusters). Right: The definition of identified cell types. (B) Bar graph of relative cell proportions by cell type, a total six subsets. (C) Gene ontology (GO) enrichment analysis of the up‐regulated biological functions in myeloid subsets in ascending order of *P* value. The black stars indicate the biological process associated with neutrophil activities. (D) Flow cytometry analysis of 16‐week‐old mouse oral mucosal tissues. FACS plots show changes of CD11b^+^Ly6G^+^ and CD11b^+^Ly6G^−^ cells in CD45^+^ cells. Right: Absolute counts and percentages of CD11b^+^Ly6G^+^ neutrophils in CD45^+^ cells (*n* = 6, two‐tail Student *t*‐test). (E) Flow cytometry analysis of mouse blood. FACS plots show changes in CD11b^+^Ly6G^+^ and CD11b^+^Ly6G^−^cells. Right: Absolute counts and percentages of CD11b^+^Ly6G^+^ neutrophils in CD45^+^ cells (*n* = 6, two‐tail Student *t*‐test). (F) UMAP shows the expression of NETosis marker gene *Padi4* in all identified cell types (gray denotes minimal expression, purple intermediate, and blue high). The red circle indicates neutrophil sub‐cluster. *Padi4* mRNA is specifically expressed in this cluster. (G) Violin plots showing the expression level of *Padi4* in neutrophil cluster between two groups. (H) IHC staining of myeloperoxidase (MPO) in mouse oral mucosal tissue sections (black triangles depict MPO^+^ cells). Right: Quantification of the percentage of MPO‐stained positive cells in sections (*n* = 6, one‐way ANOVA). Scale bars, 50 µm. Epi, epithelium; S, stroma. (I) IF staining of Ly6G (purple), CitH3 (red), and PADi4 (green) in mouse gingival tissue sections, where NETs are indicated by white triangles. (Left) Low magnification, scale bar, 50 µm; (right) higher magnification, scale bar: 25 µm. (J) Percentage of Ly6G, CitH3, and PADi4 stained area, respectively. Left: graphs showing the number of neutrophils (Ly6G^+^) per 10^5^ µm^2^ of stained tissue sections. Right: NETs are quantified by the MFI of CitH3 or PADi4 staining in oral mucosal lesions, and the ratio of CitH3 to PADi4 is calculated. All data are presented as mean ± SD.

The infiltration of neutrophils and immunopathology are recognized to be a driving force of chronic periodontal disease pathology.^[^ ^25^ ^]^ In this notion, we asked whether diabetes would promote oral mucosal immunopathology through regulating neutrophil. Through reanalyzing our previously related scRNA‐seq,^[^ ^26^ ^]^ single‐cell atlas of oral mucosal tissues (gingiva) showed that increased inflammatory cell numbers in *db/db* mice were primarily attributed to the increased presence of myeloid/granulocytes (**Figure** **2**A,B). Compared with control, gingiva of *db/db* mice revealed distinct transcriptomic signatures with a higher abundance of neutrophil and monocyte (Figure S3A–C, Supporting Information). Top upregulated pathways in myeloid subsets included pathways related to inflammation, neutrophil‐mediated immunity, and response to bacterium (Figure 2C). We also found that genes up‐regulated in disease were involved in neutrophil recruitment (*Csf1r*, *Csf3*), activation, and granulopoiesis (*Itgam/Cd11b*, *Cd68*) (Figure S3D, Supporting Information).

Consistent with transcriptomic profiles, flow cytometric analysis of gingiva from *db/db* mice revealed a significant accumulation of neutrophils (CD45^+^CD11b^+^Ly6G^+^) within the affected lesions (Figure 2D), whereas the number and proportion of other myeloid cells (CD45^+^CD11b^+^Ly6G^−^) increased modestly (Figure S4A,B, Supporting Information). Notably, neutrophils in blood and cervical lymphoid nodes of *db/db* mice were also elevated, implicating a neutrophil influx in proximity to gingival lesions detected above (Figure 2E; Figure S4C–G, Supporting Information). FACS of total cells isolated from *db/db* bone marrow confirmed an increase of neutrophils mobilization in diabetic settings (Figure S4H–J, Supporting Information).

Having established that neutrophils participate in gingival lesions of *db/db* mouse model, we next investigated whether NETs were involved in this process. For this, we analyzed the transcriptome of neutrophil cluster from scRNA‐seq data. Notably, the transcriptomic expression of peptidyl‐arginine deiminase 4 (PADi4) was mainly enriched in neutrophil cluster and significantly upregulated in gingiva of *db/db* mice (Figure 2F,G). Congruent with our scRNA‐seq data, PADi4 protein, a key enzyme for NETs,^[^ ^27^ ^]^ as well as citrullinated core histones H3 (CitH3), a marker of NETs, significantly increased in mucosal tissues from *db/db* mice (Figure S5A, Supporting Information). We further examined the spatial distribution of myeloperoxidase (MPO) and revealed a larger cover area in *db/db* gingiva, suggesting cellular externalization of MPO in diabetic gingiva consistent with NETosis process (Figure 2H). As visualized by immunofluorescent co‐localization for neutrophils (Ly6G), CitH3, and PADi4, percentage of NETosis events were increased and more positive staining for NETs were within the epithelium of mucosal lesions (Figure 2I,J).

### 2.3 Removal of NETs Alleviates Oral Mucosal Inflammation and Injury of Diabetic Mice

**Figure 3:**
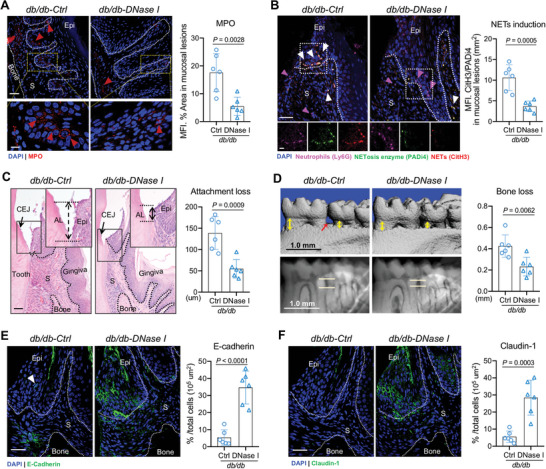
DNase‐I treatment attenuates periodontal damage and barrier disruption in *db/db* mice. (A) IF staining of MPO in gingival tissue section from *db/db* mice with or without DNase I treatment (red triangles depict MPO^+^ cells). Right: Quantification of the MFI of MPO staining in oral mucosal lesions (*n* = 6, one‐way ANOVA). Scale bars: low magnification, 30 µm; high magnification, 10 µm. Epi, epithelium; S, stroma. (B) IF staining of Ly6G (purple), CitH3 (red), and PADi4 (green) in gingival tissue from *db/db* mice with or without DNase I treatment, where NETs are indicated by white triangles. Scale bars: low magnification, 30 µm; high magnification, 10µm. Right: NETs are quantified by the MFI of CitH3 or PADi4 staining in oral mucosal lesions, and the ratio of CitH3 to PADi4 is calculated. (C) H&E staining of gingival tissues from *db/db* mice with or without DNase I treatment. The black arrowhead depicts CEJ and the distance between black lines indicates mucosal attachment loss. AL, attachment loss; CEJ, cementoenamel junction; Epi, epithelium; S, stroma. Scale bar: 50 µm. (Right) Quantification of the mucosal attachment loss (*n* = 6, two‐tail Student *t*‐test). (D) Micro‐CT visualization of periodontal bone loss of mandibles. Yellow arrows and lines indicate the distance of ABC‐CEJ in the mandible. Scale bar: 1 mm. (Right) Bone loss measurement. (E,F) IF staining of E‐cadherin (E) and Claudin‐1 (F) in gingival tissue from *db/db* mice with or without DNase I treatment. Right: Quantification of the percentage of positive E‐cadherin and Claudin‐1 cells in epithelium (*n* = 6, one‐way ANOVA). Scale bars: 30 µm. Epi, epithelium; S, stroma. All data are presented as mean ± SD.

To explore whether NETs have a role in oral mucosal inflammation and destruction induced by T2D, we used deoxyribonuclease (DNase) I to degrade NETs, as a systemic treatment. After removal of NETs, overall staining area for extracellular MPO was comparably reduced in gingival mucosa of *db/db* mice (**Figure** **3**A). Consistent with this function, gingival mucosa from DNase I‐treated *db/db* mice had significantly reduced histones citrullination and NETs‐associated enzyme expression (Figure 3B), although the effect of NETs degradation on neutrophils infiltration was less pronounced. DNase I treatment rescued mucosal inflammation of *db/db* mice and attachment loss of junctional epithelium compared with vehicle treatment (Figure 3C). Removal of NETs also led to significant protection from alveolar bone loss in these diabetic mice, revealing a pathogenic role for increased NETs in disease pathology (Figure 3D). Of note, as documented by immunostaining of E‐cadherin and Claudin‐1, DNase I treatment ameliorated the destruction of intercellular junctions at *db/db* epithelial barrier (Figure 3E,F). These results indicate that NETs contribute to the disruption of epithelial barrier and diabetes‐mediated oral mucosal inflammation.

### 2.4 Hyperglycemia Primes Oral Mucosal Neutrophils to Undergo NETosis

**Figure 4:**
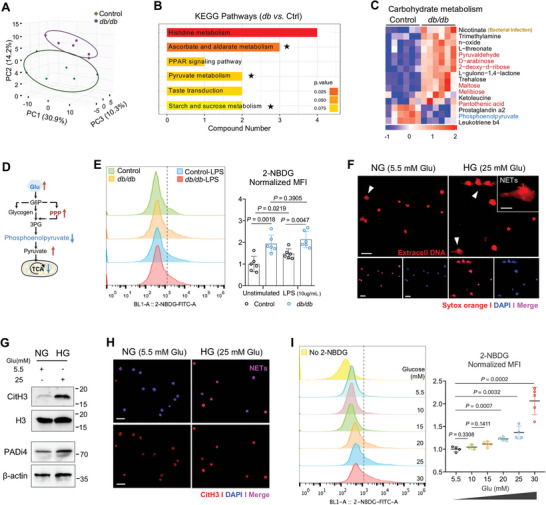
Hyperglycemia induces neutrophil extracellular traps in *db/db* mice. (A) Principal component analysis (PCA) plot in negative mode showing the distribution of differential metabolites in oral mucosa of control and *db/db* mice. (B) KEGG pathways on differential metabolites upregulated in *db/db* mice in ascending order of *p*‐value. The black stars indicate the pathways associated with carbohydrate metabolism. (C) Heatmap showing differential metabolites levels involved in carbohydrate metabolism in *db/db* gingival tissues. Red items indicate the intermediate products in carbohydrate metabolism process. (D) Schematic diagram showing the overall glucose‐associated metabolites measured in *db/db* oral mucosal tissues by LC‐MS analysis. (E) Flow cytometry analysis for glucose uptake capacity of neutrophils derived from control and *db/db* bone marrow with incorporation of glucose analog 2‐NBDG, in the presence or absence of LPS stimulation. Right: Quantification of neutrophils glucose uptake (normalized MFI of 2‐NBDG; *n* = 6; unpaired two‐tail Student *t*‐test). (F) Sytox Orange detected NETs production in vitro. The induction of NETosis is conducted on neutrophils derived from bone marrow and treated with normal glucose (5.5 mm, NG) and high glucose (25 mm, HG) concentrations for 3 h. Scale bar: 25 µm; higher magnification, scale bar: 10 µm. (G) Western blot showing the expression levels of PADi4 protein expression and citrullinated H3 in vitro. (H) CitH3 (red) and DAPI staining for NETs (purple) at 3‐h time point with 5.5 and 25 mm glucose conditions. Scale bars: 25 µm. (I) FACS analysis of glucose uptake ability in neutrophil after cultured with different glucose conditions. Neutrophils are respectively incubated with 5, 10, 15, 20, 25, and 30 mm glucose for 2.5 h and then incubated with FITC‐labelled 2‐NBDG for 30 min. Data pooled from three independent experiments, *n* ≥ 3.

We next sought to dissect mechanisms by which gingival NETs increased in T2D. Metabolic requirements for the formation of NETs have been a subject of investigation in recent years, in that glycolysis is identified as a crucial metabolic pathway in its development.^[^ ^28^ ^]^ Therefore, we aimed to determine whether hyperglycemia‐mediated metabolic pathway plays a role in oral mucosal NETs formation. As assessed by untargeted high‐resolution LC‐HRMS, principal components analysis (PCA) showed significant separation of the metabolites of the two groups (**Figure** **4**A; Figure S6A, Supporting Information), suggesting that the metabolites of *db/db* gingiva significantly differs from that of control mice. The volcano plot further revealed ≈1078 distinct metabolites (Figure S6B, Supporting Information). The functional enrichment analysis on up‐regulated metabolites suggested that most of differential pathways belonged to carbohydrate metabolism, such as ascorbate and aldarate metabolism, pyruvate metabolism, and starch and sucrose metabolic pathways (Figure 4B and Table S2, Supporting Information). Notably, heatmap for carbohydrate metabolism showed the metabolites related to glycolysis were upregulated, including pyruvaldehyde, D‐arabinose, 2‐deoxy‐d‐ribose, and maltose (Figure 4C,D), suggesting an increased glycolysis‐dependent metabolic pathway in *db/db* gingival tissues.

To define that neutrophil effector functions mainly attributed to hyperglycemia, we detected glucose level in gingival sulcus, where immune cells dynamically infiltrate or depart the gingival barrier responding to pathogenic microbes.^[^ ^7^ ^]^ As expected, gingival cervical fluid obtained from *db/db* mice displayed a higher glucose level (Figure S6C, Supporting Information). Interestingly, neutrophils derived from *db/db* bone marrow without any stimulation showed significantly increased glucose uptake compared to cells from control mice (Figure 4E), measured by enhanced incorporation of glucose analog 2‐NBDG. Compared with unstimulated group, lipopolysaccharide (LPS) treatment did not increase the glucose transport of neutrophils from *db/db* mice (Figure 4E), suggesting an impaired response of neutrophil to LPS in diabetic mouse.

Next, we challenged bone marrow‐derived neutrophils with high glucose (HG) in vitro. Nucleic acid positive staining showed increased, in association with “string‐like formations,” characteristic of NETs (Figure 4F). Western blot detected up‐regulated PADi4 protein and histones H3 citrullination level in high‐glucose cultured condition (Figure 4G). The percentage of NETosis events, evaluated by an overlap in CitH3 and nucleic DNA (DAPI), were also significantly increased (Figure 4H; Figure S6D, Supporting Information). In vitro 2‐NBDG incorporation assay revealed that neutrophils were able to translocate more glucose analog into cells at 3 h post‐high glucose stimulation in a dose‐dependent manner (Figure 4I). In addition, High‐glucose concentration exceeding 30 mm showed a negative effect on neutrophil viability (Figure S6E, Supporting Information).

### 2.5 Hyperglycemia Promotes NETs Through GLUT1‐Mediated Glycolysis

**Figure 5:**
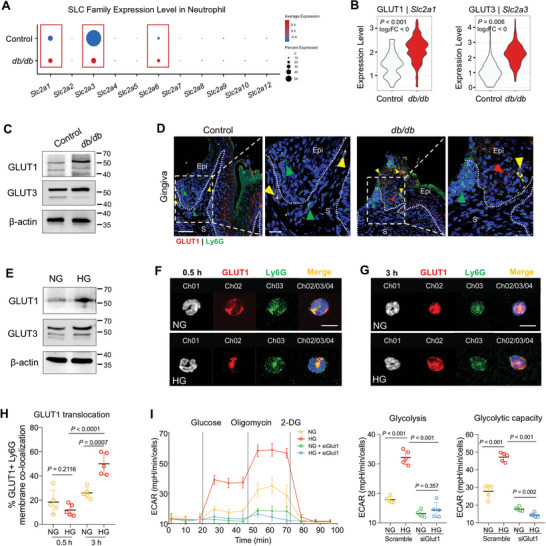
GLUT1‐mediated glycolysis primes neutrophil to undergo NETosis in T2D. (A) Bubble chart visualization of solute carrier family (SLC) mRNA expression level in neutrophil subset of mouse gingival tissue. (B) Violin plots showing transcription levels of *Slc2a1* (coding GLUT1) and *Slc2a3* (coding GLUT3) in oral mucosal neutrophils of control and *db/db* mice. (C) Western blot analyses of glucose transporter (GLUT)‐1 and GLUT3 in control and *db/db* gingival mucosa (*n* = 6). (D) IF staining of GLUT1 (red) and Ly6G (green) in mouse gingival tissue section, where co‐localized cells are indicated by yellow triangles. (Left) Low magnification, scale bar, 50 µm; (right) higher magnification, scale bar: 20 µm. Epi, epithelium; S, stroma. (E) Western blot of GLUT1 and GLUT3 in neutrophils cultured with 5.5 mm (NG) and 25 mm (HG) of glucose stimulations. (F–H) Neutrophils are stimulated with 5.5 and 25 mm of glucose for 0.5 h (F) or 3 h (G). Surface localization of GLUT1 in neutrophils is visualized by IF confocal microscopy, scale bar: 10 µm. The membrane translocations of GLUT1 represent the percentage of surface‐expressed (Ly6G co‐localized) GLUT1 out of total GLUT1 protein (*n* = 5, two‐tail Student *t*‐test). Data pooled from 3 to 4 independent experiments, and representative images (F,G) and histogram plots (H) are shown. (I) BM neutrophils are stimulated with 5.5 and 25 mm of glucose for 3 h. Small interfering RNAs (siRNAs) target to deplete *Glut1/Slc2a1* in BM neutrophils. Seahorse assay is performed to detect the ECAR (Basal extracellular acidification rate) after 5.5 and 25 mm glucose stimulations, in the presence or absence of *siGlut1*. Data pooled from three independent experiments; *n* = 5; statistical analysis by two‐way ANOVA.

To obtain insights into how hyperglycemia induces NETs by regulating glycolysis pathway, we reanalyzed scRNA‐seq data to get the expression levels of 11 glucose transporters in neutrophils. Intriguingly, the *Slc2a1* (encoding Glucose transporter 1, GLUT1), *Slc2a3* (encoding GLUT3), and *Slc2a6* (encoding GLUT6) were significantly increased in neutrophil cluster from *db/db* mice compared to that from control mice, but others were no difference (**Figure** **5**A; Figure S7A, Supporting Information). Among the aforementioned transporters, *Slc2a6* was little expressed in neutrophil compared to other two genes (Figure S7A, Supporting Information), and the gene expression level of *Slc2a3* was highly expressed but the protein level just revealed a slight elevation in *db/db* mice compared with control mice (Figure 5B,C). This finding is further supported by the presence of a few GLUT3^+^ neutrophils observed in subepithelial fibrous tissues using immunostaining (Figure S7B,C, Supporting Information). Consistent with scRNA‐seq data, the protein level of GLUT1 is significantly upregulated in *db/db* mice compared to that from control mice (Figure 5C). Moreover, immunofluorescence staining with GLUT1 and Ly6G in gingival tissues revealed an increased accumulation of GLUT1^+^ neutrophils in *db/db* mice, and they were frequently observed in proximity to severely disrupted epithelial attachment sites (Figure 5D; Figure S7D, Supporting Information).

Consistently, high glucose significantly induced the expression of GLUT1 in bone marrow‐derived neutrophils, while that of GLUT3 was not as obvious (Figure 5E; Figure S7E, Supporting Information). Immuno‐image analysis revealed a rapid expression of GLUT1 in cytoplasmic vesicles within 0.5 h of high‐glucose stimulation, followed by multitude GLUT1 translocation to the cell membrane at 3 h post‐stimulation (Figure 5F,H). When subjected to a glycolysis stress test, high glucose significantly elevated the basal glycolysis and glycolytic capacity of neutrophils (Figure 5I). We next investigated the potential effect of GLUT1 on the core glycolysis of neutrophils, through siRNA‐specific knockdown of *Slc2a1*. With confirmed deficiency of GLUT1 (Figure S7F, Supporting Information), we observed a repression in the glycolysis and glycolytic capacity following high‐glucose stimulation (Figure 5I).

### 2.6 GLUT1 Inhibition Attenuates NETs and Oral Mucosal Injury of Diabetic Mice

**Figure 6:**
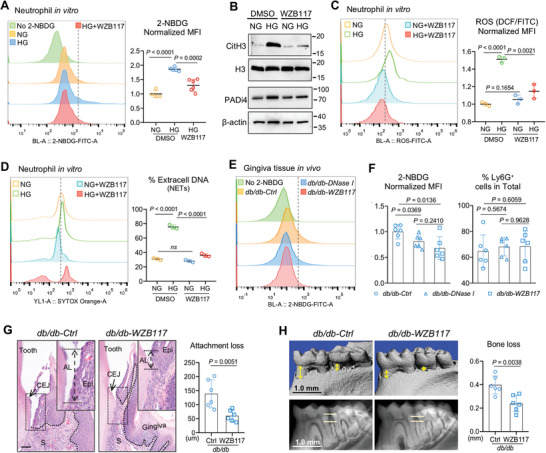
Inhibition of GLUT1 ameliorates NETs‐driven chronic inflammation at oral mucosal sites. (A) Detection of glucose uptake in neutrophils by flow cytometry (left), and bar graph (right) showing the normalized MFI of 2‐NBDG (*n* = 6). (B) Western blot analyses of PADi4 and CitH3 in neutrophils stimulated with 5.5 (NG) and 25 mm (HG) of glucose in the presence and absence of WZB117. (C) ROS production measured by FACS in the presence and absence of WZB117. Right: bar graph showing the normalized MFI of ROS/DCF (*n* = 3, two‐tail Student *t*‐test). (D) NETs formation detected by FACS of Sytox orange staining. Right: frequency of NETosis score is blindly evaluated (*n* = 3; ns, not significant as determined by ANOVA). (E) Glucose uptake of neutrophils in *db/db*‐Ctrl, DNase I‐treated *db/db*, and WZB117‐treated *db/db* oral mucosal tissues are detected by 2‐NBDG incorporation, for 1 h in vivo after intravenous injection. (F) Percentages of neutrophils in total cells (left) and the normalized MFI of 2‐NBDG (right) in *db/db*‐Ctrl, *db/db* DNase I‐treated and *db/db* WZB117‐treated mouse gingiva. Data was analyzed using one‐way ANOVA, *n* = 6. (G) H&E staining of oral mucosal tissue sections from *db/db* and WZB117‐treated *db/db* mice. The black arrowhead depicts CEJ and the distance between black lines indicates mucosal attachment loss. AL, attachment loss; CEJ, cementoenamel junction; Epi, epithelium; S, stroma. Scale bar: 50 µm. Bar graph (right) showing quantification of mucosal attachment loss, *n* = 6, Student *t*‐test. (H) Micro‐CT visualization for periodontal bone loss of mandible in *db/db* and WZB117‐treated *db/db* mice. Scale bar: 1 mm. Yellow arrows and lines indicate the distance of ABC‐CEJ in mandible. Bar graph (right) showing bone loss measurement (*n* = 6, Student *t*‐test).

Our collective data thus far demonstrated that diabetes enhanced oral mucosal lesions through hyperglycemia‐induced NETosis, with GLUT1 potentially playing a pivotal role in this phenomenon. To further validate the function of GLUT1 in NETs formation, we used a specific inhibitor, WZB117. Flow cytometric analysis of 2‐NBDG labeled cells first confirmed that WZB117 treatment reduced but did not totally block glucose transport of neutrophils in high glucose (**Figure** **6**A). Histones citrullination is critical for NET formation and has been shown to potentiate the inflammatory functions of NETs.^[^ ^29^ ^]^ As documented by western blotting, WZB117 was able to significantly reduce levels of CitH3 in high glucose, whereas expression of PADi4 was slightly diminished, suggesting the regulation of GLUT1 pathway on citrullination response but not enzymic itself during NETosis (Figure 6B). Furthermore, high glucose evoked neutrophil reactive oxygen species (ROS) production (Figure 6C), and treatment with the GLUT1 inhibitor WZB117 significantly inhibited its production. Since NET formation is partially dependent on ROS generation,^[^ ^30^ ^]^ we then evaluated NETosis in the presence of WZB117. As visualized by Sytox‐orange staining, WZB117‐treated neutrophils significantly reduced high glucose‐induced NETs formation (Figure 6D).

We further used incorporation of glucose analog 2‐NBDG to monitor the glucose transport by neutrophils in vivo. As measured by flow cytometry, systemic WZB117 treatment impaired glucose transport of gingival infiltrating neutrophils within the tissue lesions, with comparable Ly6G^+^ neutrophils among *db/db*‐WZB117, *db/db*‐DNase I and *db/db*‐Ctrl group (Figure 6E,F). Immunostaining of gingival tissues revealed minimal co‐localization of CitH3 and PADi4 in WZB117‐treated *db/db* mouse; however, WZB117 treatment did not alter neutrophil accumulation within the tissue lesions (Figure S8A,B, Supporting Information), consistent with results of gingival flow cytometry (Figure 6F). Significantly, WZB117 treatment for *db/db* mice had protection from the disruption of epithelial junction, infiltration of leukocytes, and gingival attachment loss (Figure 6G), in particular the periodontal bone loss (Figure 6H).

### 2.7 NETs Potentiate Oral Mucosal Injury and Epithelial Dysfunction of T2D Patients

**Figure 7:**
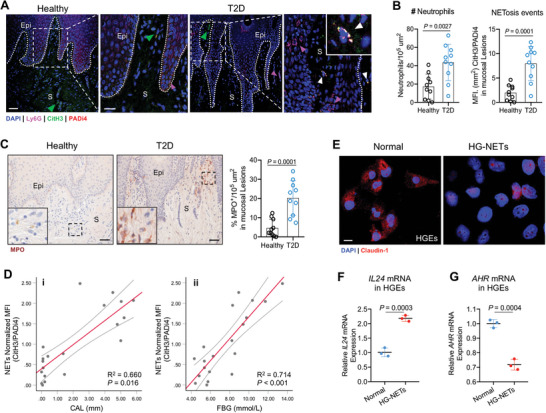
NETs potentiate oral mucosal injury and barrier disruption in T2D patients. (A) IF staining of Ly6G (purple), CitH3 (red), and PADi4 (green) in oral mucosal tissues from patients with type 2 diabetes and healthy controls. White triangles indicate NETs. (Left) Low magnification, scale bar, 50 µm; (right) higher magnification, scale bar: 25 µm. Epi, epithelium; S, stroma. (B) Left: graphs showing the number of neutrophils per 10^5^ µm^2^ of stained tissue sections. Right: NETosis events are quantified by the MFI of CitH3 or PADi4 staining in oral mucosal lesions, and the ratio of CitH3 to PADi4 is calculated. Data was analyzed using one‐way ANOVA, *n* = 10. (C) IHC staining of MPO in oral mucosal tissues from patients and healthy controls (black triangles depict MPO^+^ cells). Scale bars, 50 µm. Right: Quantification of percentage of MPO stained positive cells (*n* = 10). (D‐i) Correlation analysis of CAL (in mm) with gingival levels of NETs measured in T2D patients and healthy donors. (D‐ii) Correlation between levels of fasting blood glucose and gingival NETs measured in patients and healthy donors. Results about gingival NETs complexes are expressed as normalized MFI of CitH3/PADi4 (Figure 7B). Data was analyzed using two‐way ANOVA test, *n* = 20. (E) IF staining of Claudin‐1 (red) in human gingival epithelial (HGE) cell lines. Scale bar, 10 µm. HGEs are cultured in the absence (Normal) or presence of high glucose‐induced NETs (HG‐NETs) for 24 h. NETs formation is incubated with 25 mmol L^−1^ glucose (HG) for 3 h. (F) Expressions of *IL24* mRNA in HGEs are measured by *qPCR*, normalized to *GAPDH*. (G) Expressions of *AHR* mRNA in HGEs are measured by *qPCR*, normalized to *GAPDH*. Data are representative of three independent experiments. Graphs show the mean ± SD. Data was analyzed using unpaired two‐tailed Student *t*‐test, *n* = 3.

We further evaluated the clinical relevance of enhanced NETosis in murine gingival neutrophils. Consistent with NETosis in vivo, co‐localization of CitH3, PADi4, and Ly6G staining was increased in gingival samples from T2D patients, along with more Ly6G^+^ neutrophils infiltrated in proximity to the injured epithelium (**Figure** **7**A,B). Compared with healthy donors, at the mucosal site of disease, there was a significantly enhanced diffuse extracellular staining for MPO (Figure 7C). We next quantified the staining results of CitH3/PADi4, as a surrogate for gingival levels of NETs. In fact, the levels of gingival NETs were strongly correlated with the severity of mucosal destruction (measured in clinical attachment loss, CAL) at the specific area where the clinical sample was collected (Figure 7D‐i). In addition, hyperglycemic levels in patients (measured in fasting blood glucose, FBG) was significantly associated with a higher level of gingival NETs complexes (Figure 7D‐ii).

Staining of gingival samples for CitH3 and MPO indicate that neutrophil NETs and epithelial cells are in close proximity in mucosal lesions (Figure 7A,C), allowing for potential interactions. When high glucose‐induced NETs were used to treat human gingival epithelial (HGE) cell line, the epithelial integrity markers Claudin‐1 was decreased (Figure 7E), and the epithelial injury markers *Interleukin* (*IL*)*‐24* ^[^ ^31^ ^]^ was increased in HGEs (Figure 7F). The junctional epithelium with large intercellular spaces and decreased levels of Claudin‐1 has been considered a relatively weak barrier to invading bacteria.^[^ ^32^ ^]^ As determined by qPCR (Figure 7G), NET‐treated HGEs also reduced gene transcription of *aryl hydrocarbon receptor* (*AHR*), which is a critical player in mucosal homeostasis and microbial signaling.^[^ ^33^ ^]^

## 3 Discussion

Numerous theories have been put up to explain the relationship between diabetes and increased risks for periodontitis in gingival mucosa, and many studies focus on the possibly impaired neutrophil functions.^[^ ^1^, ^34^, ^35^ ^]^ Here, we demonstrated a causative link between NETs and gingival barrier immunopathology induced by T2D. Furthermore, hyperglycemia was identified as a driving force of NETosis, in which uncontrolled NETs formation was evoked by GLUT1‐mediated glycolysis, thus leading to tissue inflammation and epithelial barrier disruption.

Diabetes is a known risk factor for periodontitis or wound healing, which has been associated with impaired metabolic and exaggerated immunological responses.^[^ ^34^, ^36^, ^37^ ^]^ Indeed, hyperglycemia in T2D instigates chronic inflammation, at least partly through alterations in neutrophil production, activation, and function. Hyperglycemia has been reported to drive the production and mobilization of neutrophils from the bone marrow and thus contributing to the pathogenesis of diabetic complications.^[^ ^38^, ^39^ ^]^ After their release into the blood circulation, the neutrophils can be recruited to peripheral tissues in response to infection and/or inflammation.^[^ ^40^ ^]^ Takanori et al., revealed that enhancing chemokines expression in gingiva normalized diabetes‐induced delays in neutrophils recruitment and periodontitis.^[^ ^34^ ^]^ It seems that in T2D, hyperglycemia‐induced neutrophil migration to pathologic sites, serve as a major cause for gingival inflammation. Notably, protection from gingival mucosal injury and bone loss in DNase I‐treated *db/db* mice occurred, despite comparable numbers of neutrophils within the tissue microenvironment. NETs formation is linked to both diabetic inflammation and impaired wound healing.^[^ ^41^, ^42^ ^]^ NETs evolved to protect the host against microbial infections and are formed by a web‐like structure of DNA that is decorated with antimicrobial effectors.^[^ ^43^, ^44^ ^]^ Due to their potent inflammatory functions, NETs cause tissue damage and aggravate inflammatory diseases. Their formation has been documented in tissue biopsies of patients with chronic periodontitis.^[^ ^45^, ^46^ ^]^ A delicate metabolic balance exists in gingival infiltrated neutrophil, serving a physiologically protective mechanism and promoting NETs formation for microbial clearance. However, onset of hyperglycemia disrupts the metabolic balance and hyperactivates neutrophils, which lead to their over‐functioning. For instance, diabetic microenvironment impedes phagocytic ability in neutrophils, which may be compensated by NETs‐mediated killing.^[^ ^47^ ^]^

We further determine that hyperglycemia primes gingival neutrophils to undergo NETosis in mucosal lesions. Higher levels of glucose and advanced glycation end products (AGEs) in diabetes can induce neutrophil activation and subsequently escalated oxidative stress via RAGE‐ERK1/2 pathways.^[^ ^1^ ^]^ Elevation of ROS may be one of the reasons for the constitutive production of NETs in hyperglycemic conditions.^[^ ^48^ ^]^ Persistent hyperglycemia is often accompanied by increased production of free radicals or impaired antioxidant defenses. It is noteworthy that high neutrophil count in diabetes is in keeping with an increased oxidative stress triggered by the high levels of hyperglycemia observed in clinical cohort.^[^ ^49^ ^]^ Glucose also induced Nicotinamide adenine dinucleotide phosphate (NADPH) oxidase required by neutrophil for NETs formation.^[^ ^50^ ^]^ Neutrophils in T2D have a higher concentration of intracellular calcium and on the other hand, calcium flux is required for NETs formation. Increase in calcium flux elevates PADi4 levels which mediate histone citrullination. Other studies through pre‐clinical and clinical models demonstrate that hyperglycemia activates neutrophils constitutively but impedes their response to infections.^[^ ^22^, ^48^ ^]^ We found that the response of neutrophil to LPS was impaired in diabetic mouse. Earlier study indeed have shown that hyperglycemic conditions in T2D induced constitutive NETosis and further neutrophils failed to form NETs in response to LPS.^[^ ^48^ ^]^ Moreover, it was observed that in T2D, neutrophils on treatment with IL‐6, LPS and TNF‐α did not form any extended NETs.^[^ ^50^, ^51^ ^]^ Therefore, NETosis may not be induced by LPS or oral microbial dysbiosis in this model, which can also indirectly affect neutrophil functioning. Moreover, hyperglycemia might be the consequence of marked inflammation resulting from the stress of periodontal destruction, and as such, it represents just the tip of the iceberg.^[^ ^49^, ^52^ ^]^

Hyperglycemia‐induced epithelial dysfunction also has been extensively studied in recent years. Hyperglycemia resulted in decreased expression of ZO‐1 and occluding and a downregulation in epithelial connexin,^[^ ^53^ ^]^ suggesting a link between barrier dysfunction and increased risk for respiratory infection in diabetes. Intestinal epithelial cells exposed to hyperglycemia reprogrammed the transcriptome of tight junction proteins, leading to enhanced permeability and infection susceptibility.^[^ ^54^ ^]^ In our work, hyperglycemia‐induced NETs had similar effects on epithelial barrier at oral mucosa, evidenced by profound leukocytes infiltration and attachment loss of gingiva with disruption of junctional epithelium. Bacterial invasion into gingival mucosa and persistent infection are major events that lead to periodontitis. Increased barrier permeability allows the translocation of microbial contents to gingival tissues and systemic circulation, contributing to neutrophils influx and chronic inflammation.

It is extremely difficult to determine whether NETs have a direct or indirect role in the induction of epithelial barrier disruption in T2D. NETs contain a plethora of pleiotropic inflammatory agents with broad functionalities. Even when considering solely the role of cytotoxic histones, their functions are pleiotropic, and their known receptors are not selective. Sensing of double‐stranded DNA (dsDNA, another component of NETs) by AIM2 in epithelial cells is crucial to mediate protection against invading pathogens,^[^ ^55^ ^]^ establishing a connection to epithelial homeostasis. Therefore, it is plausible that more than one factor/mechanism is involved in NET‐associated induction of epithelial barrier disruption. A recent study confirms that NETs trigger IL‐17 immunity to promote oral mucosal immunopathology. NETs are also suggested to abet the barrier immunopathology in ulcerative colitis, by promoting coagulation cascades.^[^ ^56^ ^]^ Perhaps, neutrophils in T2D do not mediate inflammation simply by their increased numbers and activities but also by their absence from the tissue.^[^ ^57^ ^]^

The regulation of cellular metabolic pathways is a critical determinant of immune function, a field that has gained renewed appreciation in recent years. Hyperglycemia in T2D significantly reprograms neutrophil metabolism and impedes its effector. In diabetic subjects, decreasing glucose level by sodium‐glucose cotransporter (SGLT) inhibitor lower neutrophil inflammatory response, as neutrophils preferentially utilize glucose from glycolysis as an energy source.^[^ ^58^ ^]^ Primed/activated neutrophils increase GLUT receptors on their surfaces associated with more glucose uptake.^[^ ^59^, ^60^ ^]^ Conversely, disruption of GLUT1 and glucose uptake in hematopoietic progenitors cause a reduction in myelopoiesis and thus the counts of circulating neutrophils. Prior study demonstrated the metabolic requirement of NETs formation where, chromatin condensation was glucose‐independent and however, glucose was required for chromatin release during NETosis.^[^ ^61^ ^]^ When treated with hexokinase inhibitor, 2‐deoxyglucose (2‐DG), neutrophils reduced NETs formation in response to glucose.^[^ ^48^ ^]^ Inhibition of GLUT1 protects *db/db* mice from NETs‐mediated inflammatory bone loss, implying that GLUT1 contributes to periodontitis in individuals with hyperglycemia and/or compromised metabolic function, at least partly, through NETosis. Metabolic pathways that regulate glycolysis and energy supply are tightly linked to the capacity of NETs production,^[^ ^62^ ^]^ for example those that regulate NADPH levels.^[^ ^63^ ^]^ Recently, phosphofructokinase‐1 liver type (PFKL) was identified as a modulator of glycolysis in neutrophils,^[^ ^64^ ^]^ which negatively regulates the glycolytic flux and tunes NADPH oxidase‐dependent NETosis. Interestingly, cellular glycolysis also underlies the induction of trained innate immunity,^[^ ^65^ ^]^ including trained neutrophils; these trained cells populate oral and nonoral tissues, thereby not only exacerbating periodontitis but also increasing the severity of inflammatory comorbidities, such as diabetes.^[^ ^37^, ^66^ ^]^

The major limitation of our study is systemic treatment with DNase rather than a topical use. Although DNase I mainly focuses on removal of NETs, we could not rule out its pharmacological effect on other immune cells, such as the activated inflammasome in macrophage. Another limitation is that our results do not completely rule out the effect of other potential metabolic pathways, such as stored glycogen and *β*‐oxidation, in mediating NETs formation. In addition, it is extremely difficult to determine whether NETs have a direct role in reduction of tight and adherent junctions. Future studies of the functional role of common polymorphisms in *SLC2A1* locus are necessary to define whether glucose metabolic disorder underlies susceptibility. The mouse model of neutrophil‐specific PADi4/GLUT1 knockout will be warranted to validate NETs and/or GLUT1 as a therapeutic targeting of disease.

In conclusion, this work demonstrates a novel immuno‐metabolic link between hyperglycemia and oral mucosal immunopathology, which is mediated through GLUT1‐dependent NETs formation. Targeting neutrophil metabolism could be a promising therapeutic strategy in the prevalent periodontal diseases in diabetic patients. While NETs are well‐described features of T2D or periodontitis,^[^ ^23^, ^67^, ^68^ ^]^ the pathogenesis of NETs in the process of tissue destruction may reflect mechanisms shared between mucosal infection and periodontitis in diabetes.

## 4 Experimental Section

### Experimental Model and Subject Details

Male C57BKS‐*db/db* (C57BL/KsJ‐Lepr*^db^*/lepr*^db^*) (*db/db* group) and C57BKS*^+/lepr^*‐*db/m* (Control group) mice were purchased from GemPharmatech LLC. (Nanjing, China). C57BKS‐*db/db* mice developed spontaneous hyperglycemia at 8 weeks of age and were widely accepted as an animal model of T2D. They were examined at 5–16 weeks of age and euthanized at 16 weeks of age.

For high‐fat diet/streptozotocin (STZ)‐induced T2D mouse model, male C57BL/6J mice were given an intraperitoneal injection of either STZ (STZ; Sigma‐Aldrich, Shanghai, China) or saline with a low dose (40 mg kg^−1^ day^−1^) after 4‐week high‐fat diet. After five to eight injections, mice with non‐fasting blood glucose levels ≥250 mg dL^−1^ were chosen for the study.

All experiments involving animal subjects were performed in accordance with the Subcommittee on Research and Animal Care (SRAC) of Sichuan University‐approved protocols (WCHSIRB‐D‐2021‐547). Animals were handled in the State Key Laboratory of Oral Diseases at Sichuan University, under standard housing conditions with a 12‐h light‐dark cycle in pathogen‐free facilities. Five‐week‐old male mice were used in all experiments unless otherwise noted. Blood glucose and weight measurements were recorded every 2 weeks from 6 weeks old.

### Micro‐Computed Tomography (Micro‐CT)

Mouse mandibles were dissected at 8 or 16 weeks of age (at least six mice for each age group) for quantitative analysis of the alveolar bone. Hemi‐mandible were then examined by a micro‐CT system (µCT50, SCANCO Medical, Bruettisellen, Switzerland) with a spatial resolution of 8 µm as previously described.^[^ ^26^ ^]^ All images of these mandibular samples were captured by 3D reconstruction to analyze the periodontal bone loss. The distance between the cementoenamel junction and alveolar bone crest (CEJ‐ABC distance) was measured at one predetermined site (the mesial part of the first molar). In addition, the X‐ray observation of bone resorption between two neighboring teeth also showed the severity of periodontal bone loss between different groups, through measuring the vertical CEJ‐ABC distance. Individual mouse scores were then averaged within experimental groups for final loss levels. Measurements were expressed in mm^2^.

### Histological Analysis

All samples from experimental animals were collected at designated ages. Mandible samples were fixed in 4% PFA at 4 °C for 24 h, and then decalcified in 10% ethylenediaminetetraacetic acid (EDTA) for 3–4 weeks. Briefly, 5 µm thick paraffin sections were deparaffinized in xylene and rehydrated in graded ethanol series.

For hematoxylin‐Eosin (HE) staining, sections were stained with hematoxylin for 10 s, followed by eosin for 15 s. After staining, sections were dehydrated through 100% ethanol and cleared in xylene.

Immunofluorescence (IF) staining was performed as previously described.^[^ ^69^, ^70^ ^]^ Primary antibodies were used at a certain dilution (anti‐CD45, BD Bioscience 561018, 1:500; anti‐Ly6G, Biolegend 108412, 1:200; anti‐CitH3, Abcam ab281584, 1:1000; anti‐PADi4, Abcam ab214810, 1:1000; anti‐MPO/Myeloperoxidase, Santa Cruz sc‐52707, 1:100; anti‐E‐cadherin, Santa Cruz 8426, 1:100; anti‐Claudin‐1, Santa Cruz 166338, 1:100; anti‐GLUT1, Abcam ab115730, 1:5000; anti‐GLUT3, Abcam ab191071,1:1000) at 4 °C overnight. Secondary staining was performed with Alexa Fluor 488/550/647‐conjugated Anti‐Rabbit or Anti‐Mouse secondary antibodies (1:200, Invitrogen, USA) for 1 h at 37 °C. Nuclei were counterstained with 4′,6‐diamidino‐2‐phenylindole (DAPI) (1:1000) for 1 h at room temperature. Images were captured by a laser scanning confocal microscope (Olympus FV3000).

For immunohistochemical (IHC) staining, sections were treated with 3% hydrogen peroxide for 15 min to reduce endogenous peroxidase activity. Then slices were immersed in sodium citrate buffer for 10 min at 90 °C for antigen retrieval and blocked with 5% BSA for 30 min afterward. The primary antibodies anti‐MPO (1:100; sc‐52707; Santa Cruz, USA), anti‐E‐cadherin (1:100; sc‐8426; Santa Cruz, USA) and anti‐Claudin‐1 (1:100; sc‐166338; Santa Cruz, USA) were incubated overnight at 4 °C. Goat anti‐mouse secondary antibody was applied for 1 h the following day and then the sections were detected by AEC Staining Kit (Boster Biological Technology).

### Single‐Cell RNA Sequencing

The single‐cell RNA sequencing (scRNA‐seq) data was obtained from our previous publication^[^ ^26^ ^]^ about gingival mucosal map of control and *db/db* mice. Initial sequencing data were compared to the mouse genome mm10 and folded the unique molecular identifier (UMI) with CellrRanger (version 3.1, 10× Genomics) software to obtain a single‐cell gene expression matrix. The expression matrix was imported into the Seurat package (version 4.0) for further analysis. Genes expressed in less than three cells were deleted, mitochondrial genes > 25%, and cells with genes < 300 were filtered out. The FindVariableGenes function in the Seurat package was used to select variable genes, and then principal component analysis (PCA) was performed, and UMAP dimensionality reduction and visualization were performed based on PCA results. According to the specific genes of different subgroups, cell types of different sub‐clusters were annotated. Gene ontology (GO) enrichment analysis was performed using Enrichr or Metascape.org on the top 200 differentially expressed genes (adjusted *p* < 0.05 by Wilcoxon Rank Sum test).^[^ ^71^ ^]^ GO terms shown were enriched at FDR < 0.01. Differential gene expression was evaluated by DESeq2 independent statistical methods. Differentially expressed genes [false discover rate (FDR) < 0.01] were considered for further analyses, based on results from DESeq2.

### Flow Cytometry for Tissues

Single‐cell suspensions from gingival tissues were prepared as previously described.^[^ ^26^ ^]^ Briefly, gingival tissues were subjected to mechanical dissociation and digested with 2 mg mL^−1^ collagenase II (Worthington Biochemicals) and 1 mg/mL DNase I (Sigma) solution for 1 h. Homogenates were passed through 70 µm cell strainer. Cells were washed in 0.04% non‐acetylated BSA.

For blood cells, fresh blood from mice was collected into tubes containing red blood cell lysis buffer (v/v, 1:5), splitting at room temperature for 10 min. After centrifuged, cells washed in PBS were passed through 70 µm cell strainer for 2 times.

Then, the single‐cell suspensions described above were incubated with mouse serum (Jackson Immuno‐Research Lab) and fluorochrome‐conjugated antibodies against surface markers in PBS with 2.5% FBS, for 20 mins at 4 °C in the dark, and then washed. Dead cells were excluded with Live/Dead fixable dye (Amcyan, 1:100, Invitrogen). Anti‐mouse antibodies used for staining were from Rabbit and purchased from Thermo Scientific and BioLegend, including CD45 (APC/Cyanine7, BioLegend 103115), CD11b (FITC, BioLegend 101205), Ly6G (APC, BioLegend 108411). The single‐dye tubes were prepared for adjusting the compensation curve in the process of operation. Cell acquisition was performed in LSRFortessa (BD Biosciences) flow cytometer, and further analyzed by FlowJo software (Tree Star). Three independent repeated experiments were conducted.

### Granulopoiesis Measurement

For prepared cells from cervical lymph node and mice bone marrow (BM), a single‐cell suspension was generated by mashing samples through a 70 µm filter and washed in PBS. BM cells from femur and tibia were stained with anti‐Ly6G and anti‐CD11b antibodies; lymph node cells from proximity to gingival immunopathology tissues were stained with anti‐CD45, anti‐Ly6G, and anti‐CD11b antibodies and analyzed by flow cytometry analysis. All samples were analyzed using a FACS Fortessa cytometer (BD Biosciences). Data analysis was performed using FlowJo software (Tree Star).

### Western Blot Analysis

Total protein was extracted according to the manufacturer's specifications (SAB, USA). The samples were subjected to 5% (w/v) sodium dodecyl sulfate‐polyacrylamide gel electrophoresis (SDS‐PAGE) and transferred to nitrocellulose membranes by electro‐blotting. The membranes were incubated overnight at 4 °C with primary antibodies (anti‐CitH3, Abcam ab281584, 1:1000; anti‐Histone H3, Abcam ab213224, 1:1000; anti‐PADi4, Abcam ab214810, 1:1000; anti‐GLUT1, Abcam ab115730, 1:5000; anti‐GLUT3, Abcam ab191071,1:1000; anti‐β‐actin, Proteintech, 1:5000) then with secondary antibodies for 1 h at room temperature. The signals were detected by electrochemiluminescence using a Bio‐Rad system (Bio‐Rad Laboratories, Shanghai, China). For quantitation, citrullinated proteins were normalized to the levels of the total core histones H3 protein, and other proteins were accomplished by normalizing to β‐actin.

For in vitro experiments with different stimulations, neutrophils were lysed by lysis buffer supplemented with protease inhibitor cocktail. Lysates were separated by SDS‐PAGE and transferred to polyvinylidene difluoride membranes. After incubation with primary and secondary antibodies, protein bands were detected using an enhanced chemiluminescence detection system.

### DNase I Treatment

As previously described,^[^ ^46^ ^]^ *db/db* mice of 6 weeks of age were i.p. injected with either 400 U of DNase I (Sigma, Roche, Shanghai, China) in 100 mL of 0.9% NaCl or 0.9% NaCl, twice a week for 10–12 weeks. Mandibles were harvested for further experiments and bone loss measurements were taken as described under “bone loss measurements.”

### Untargeted LC‐HRMS

The gingival tissues separated from mice were quickly frozen in liquid nitrogen immediately after dissection. Then the tissues were cut on dry ice into a 2 mL tube. The tissue samples with 200 µL of H_2_O and five ceramic beads were homogenized. Then 800 µL methanol/acetonitrile (1:1, v/v) were added to homogenized solution for metabolite extraction. The mixture was centrifuged for 20 min and supernatant was dried in a vacuum centrifuge. For LC‐MS analysis, the samples were re‐dissolved in 100 µL acetonitrile/water (1:1, v/v) solvent and centrifuged, then the supernatant was injected.

Analyses were performed by untargeted LC‐HRMS as previously described.^[^ ^28^ ^]^ Briefly, samples were injected via Thermo Vanquish UHPLC and separated over a reversed‐phase Thermo HyperCarb porous graphite column. For LC gradient, the mobile phase consisted of solvent A (water/0.1% FA) and solvent B (ACN/0.1% FA). The gradient was the following: 0–1 min 1% B, increase to 15% B over 5 min, continue increasing to 98% B over 5 min, hold at 98% B for 5 min, re‐equilibrate at 1% B for 5 min. Thermo IDX tribrid mass spectrometer was operated in both positive and ion mode, scanning in ddMS2 mode with an AGC target of 2 × 10^5^ for full scan, 2 × 10^4^ for ms2 scans. Calibration was performed prior to analysis using the PierceTM FlexMix Ion Calibration Solutions. Untargeted differential comparisons were performed using Compound Discoverer 3.0 to generate a ranked list of significant compounds with tentative identifications from BioCyc, Kyoto Encyclopedia of Genes and Genomes (KEGG), and internal compound databases. Compound identification of metabolites was performed by comparing of accuracy m/z value (<10 ppm), and MS/MS spectra with an in‐house database established with available authentic standards. After sum‐normalization, the processed data were analyzed by R package, where it was subjected to multivariate data analysis, including Pareto‐scaled principal component analysis (PCA). Student's *t*‐test was applied to determine the significance of differences between two groups of independent samples. VIP > 1 and *p* < 0.05 were used to screen significant changed metabolites.

### Neutrophil Isolation and Culture

Neutrophils were isolated as described previously.^[^ ^46^ ^]^ Briefly, bone marrow cells were flushed out from femurs and tibiae of 8‐ to 10‐week‐old mice with RPMI 1640 Medium (Gibco) supplemented with 2 mm EDTA, 20% heat‐inactivated fetal bovine serum (FBS), and 1% penicillin‐streptomycin. Neutrophils were isolated from total bone marrow cells by density gradient centrifugation. Dilute Percoll solution (100%) with 10× PBS to three different densities of Percoll working solutions: 70%, 62%, and 50%. Slowly and carefully add 2 mL of 70%, 62%, and 50% Percoll working solution along the tube wall and 1 mL RPMI‐1640 resuspended suspension was added on the top at 25 °C, 400 g for 30 min without break on Percoll. The purity of the neutrophils was above 95% as determined by flow cytometry analysis. Then neutrophils were washed with PBS and suspended in RPMI 1640 supplemented with 10% heat inactivated FBS.

For in vitro experiment, BM‐derived neutrophils were cultured with RPMI 1640 supplemented with 5.5 mm (normal glucose, NG) and 25 mm (high glucose, HG) glucose. RPMI 1640 containing 5.5, 10, 15, 20, 25, and 30 mm glucose was prepared for the glucose concentration gradient conditioned medium.

For NETs formation, BM‐derived neutrophils (1 × 10^6^) were seeded in RPMI 1640 supplemented with 3% FBS and stimulated with 25 mm high glucose for 4 h. Afterward, the culture medium was removed, and NETs were incubated with the restriction enzymes BseRI, PacI, NdeI, and AfII (New England Biolabs) for 1h. Supernatants containing the NETs fragments were collected and centrifuged 10 min at 1, 000 g to remove remaining cell debris. NETs were verified by staining with Sytox orange (Thermo Fisher Scientific, S11368).

### Human Gingival Epithelial Cells Culture

Human gingival epithelial (HGE) cell lines were purchased from Otwo Biotech (HTX2651). HGEs were cultured in DMEM medium with 1% antibiotic solution and 10% FBS. Cells were treated with 200 ng mL^−1^ or 300 ng mL^−1^ NETs (supernatants containing the NETs fragments described above) for 24 h. The normal group was only cultured in medium without high glucose‐induced NETs.

### Glucose Uptake Measurement

Total cells from bone marrow of control and *db/db* mice, respectively (Figure 4E), were seeded in six‐well flat bottom plates and cultured with 5.5 mm glucose medium in the absence or presence of lipopolysaccharide (LPS) stimulation, at 37 °C for 2.5 h. The cells were washed with PBS and 100 µL 2‐[N‐(7‐nitrobenz‐2‐oxa‐1,3‐diazol‐4‐yl) amino]‐2‐deoxy‐D‐glucose (2‐NBDG, 100 µM in PBS) was added. Cells were incubated at 37 °C for another 30 min. Staining with anti‐mouse Ly6G antibodies (APC, BioLegend a108411, 1:200), the glucose uptake by neutrophils was detected by FACS.

BM‐derived neutrophils were seeded in six‐well flat bottom plates and stimulated with conditioned medium at 37 °C for 2.5 h (Figure 4I and 6A). The cells were washed with PBS and 100 µL 2‐NBDG (100 µM in PBS) was added. Cells were incubated at 37 °C for another 30 min. Staining with anti‐mouse Ly6G antibodies, the glucose uptake by neutrophils was detected by FACS.

For glucose uptake measurement in gingiva‐infiltrating neutrophils (Figure 6E), *db/db* mice were injected i.v. with 250 µL 2‐NBDG (2.5 mm in PBS). Thirty mins later, gingival tissues were subjected to mechanical dissociation and digested with 2 mg mL^−1^ collagenase II (Worthington Biochemicals) and 1 mg mL^−1^ DNase I (Sigma) solution for 1 h. Homogenates were passed through 70 µm cell strainer. Cells were washed in 0.04% non‐acetylated BSA. Anti‐mouse Ly6G antibodies were used for staining gingiva neutrophils. Gingiva infiltrating neutrophils were analyzed for glucose uptake by flow cytometry.

### NETosis Assay

BM‐derived neutrophils were incubated with conditioned medium (5.5 mm or 25 mm glucose) for 3h. The cells were fixed and permeabilized and NET formation was measured by staining with Sytox Orange or an anti‐CitH3 polyclonal antibody (Figure 4F,H). The secondary antibody was Alexa Fluor 550‐conjugated Anti‐Rabbit secondary antibodies. Staining was visualized using a confocal microscope (Olympus FV3000). Additionally, cells were collected and stained with Sytox Orange at 37 °C for 20 min. NETosis assay was detected by FACS (Figure 6D).

### Measurement of Glucose Level in Gingival Crevicular Fluid

Before sample collection, glucose level in the gingival crevicular fluid of two groups of mice were measured and recorded by the Glucose meter, with a measurement accuracy of 0.1 mm. The gingival crevicular fluid was aspirated by a disposable capillary suction tube under the visual field, ≈1/4 of which was enough.

### Fluorescent Immunocytochemistry (ICC) Assay

BM‐derived neutrophils were incubated with 5.5 mm or 25 mm glucose medium for 3h. The cells were fixed (4% PFA), permeabilized, and stained with primary antibody (anti‐GLUT1, Abcam ab115730, 1:10000; anti‐Ly6G, Biolegend 108412, 1:200) at 4 °C overnight. The secondary antibody used was Alexa Fluor 550‐conjugated Anti‐Rabbit secondary antibodies. Staining was visualized using a confocal microscope (Olympus FV3000).

### siRNA‐Slc2a1(Glut1) Treatment and Analysis

Small interfering RNA (siRNA) targeting mouse *Glut1* was designed and used to block *Glut1* in BM neutrophils (5′‐GGAATTCAATGCTGATGATGA‐3′, 5′‐ TCATCATCAGCATTGAATTCC‐3′, GenePharma Co.). Non‐targeted siRNA as a negative control (5′‐TTCTCCGAACGTGTCACGT‐3′, 5′‐ACGTGACACGTTCGGAGAA‐3′) was used in wells not transfected with *Glut1* siRNA. Normal saline‐treated with diethyl pyrocarbonate (DEPC, Sigma‐Aldrich Corp. Ltd., MO, USA.) was used to dissolve siRNA to reach a 20 µmol L^−1^ concentration. The transfection mixture per well was prepared using 5 µL Endofectin (GeneCopoeia Inc., USA) and 5 µL *Glut1* siRNA or noncoding siRNA.

### Seahorse Metabolic Assay

BM‐derived neutrophils from mice were isolated and plated on poly‐lysine coated Seahorse culture plates (5 × 10^6^ cells per well) in RPMI 1640 without glucose. After being stimulated with conditioned medium at 37 °C for 3 h, the cells were analyzed using a Seahorse XFe24 Analyzer (Agilent). EACR (Basal extracellular acidification rate) was detected in the presence of glucose (5.5 mm or 25 mm), oligomycin (2 mm), 2‐deoxyglucose (100 mm) to obtain maximal and control EACR values.

### Quantitative Real‐Time PCR

RNA was extracted from neutrophils or HGEs using RNeasy kits. Complementary DNA was synthesized by SuperScript III First Strand Kits. Quantitative real‐time PCR (qPCR) was performed with the PerfeCTa SYBR Green FastMix and analyzed on an ABI 7300 real‐time instrument. Primers (*Slc2a1*, *Slc2a2*, *Slc2a3*, *Slc2a4*, *IL24*, *AHR*) were obtained from QuantiTect Primer Assays and details were shown in Supporting Information. The expression of each gene was normalized to that of *Gapdh/GAPDH*.

### GLUT1 Inhibitors for In Vitro and In Vivo Studies

GLUT1 inhibitors (WZB117, 10 mm) used for the in vitro experiments were incubated with BM‐derived neutrophils 30 min before stimulation. The stock solution of WZB117 was made by dissolving in DMSO, and further diluted using media to make working concentrations.

For in vivo experiments, WZB117 (10 mg kg^−1^ body weight) was dissolved in 100 mL of PBS/DMSO solution (1:1, v/v). *Db/db* mice of 6 weeks of age were given intraperitoneal injection with either PBS/DMSO vehicle or compound WZB117 daily for 10 weeks.

### ROS Measurement

BM‐derived neutrophils were incubated with conditioned medium for 3h. Then, cells were collected and incubated with DFC/FITC at 37 °C for 30 min. The ROS production was measured by flow cytometry analysis.

### Human Oral Mucosal Samples

Gingival sections from T2D patients and healthy individuals (donors) were collected from clinical patients visiting West China Hospital of Stomatology, Sichuan University. All trials were conducted in conformance with the ethical guidelines (WCHSIRB‐D‐2022‐250). As previously described,^[^ ^26^ ^]^ inclusion criteria for diabetic mellitus group were: a) diagnosis of T2D >1 year (according to the WHO Diabetes Diagnostic Criteria); b) hemoglobin A1c (HbA1C) level >6% and no serious complications (such as infection, diabetic foot, nephropathy, and retinopathy); c) visible signs of gingival inflammation (a mean clinical attachment loss (CAL) > 1 mm and at least four teeth with a periodontal probing depth (PD) > 4 mm) including erythema/edema, and bleeding upon probing. All participants were deemed systemically healthy based on detailed medical history and select laboratory work up in donor group. Inclusion criteria for donor/healthy group were: 18 years of age and over, a minimum of 20 natural teeth, and in good general health. Exclusion criteria were: history of Hepatitis B or C, history of HIV, prior radiation therapy to the head or neck, active malignancy except localized basal or squamous cell carcinoma of the skin, treatment with systemic chemotherapeutics or radiation therapy within 3 years, pregnant or lactating, >3 hospitalizations in the last 3 years, autoimmune disorder such as Lupus, Rheumatoid Arthritis, etc. Additional exclusion criteria included the use of any of the following in the 3 months before study enrollment: systemic (intravenous, intramuscular, or oral) antibiotics, oral, intravenous, intramuscular, intranasal, or inhaled corticosteroids or other immunosuppressants, cytokine therapy, methotrexate, or immunosuppressive chemotherapeutic agents, large doses of commercial probiotics (greater than or equal to 10^8^ colony‐forming units or organisms per day), or use of tobacco products (including e‐cigarettes) within 1 year of screening. In addition to systemic screening, oral health was assessed and only participants with no soft tissue lesions, no signs/symptoms of oral/dental infection, and no/minimal gingival inflammation were considered for the health group.

Participants received detailed intraoral soft tissue and periodontal examination, which included full mouth PD and CAL (evaluation of bone destruction) and bleeding on probing (BOP, evaluation of mucosal inflammation). All surgery for obtaining gingival tissues (≈2 × 1 × 2 mm^3^) was conducted with informed consent of patients. Donor/health group samples were obtained from individuals during extraction or crown lengthening surgery, who met criteria for oral health and in areas without BOP and with PD < 3mm. T2D group samples were harvested at the buccal or lingual sites representing the highest CAL during periodontal surgery. All biopsies were subjected to paraffin embedding and stained for Ly6G/CitH3/PADi4 and MPO. Detailed information about all samples are provided in Table S1 (Supporting Information).

### Statistical Analysis

All data were expressed as mean ± standard deviation (SD). Statistical analyses were performed using GraphPad Prism software (version 8.0.0; GraphPad Inc). After confirming normality, data were analyzed with two‐tailed unpaired Student's *t‐*test (comparison of only two groups) or one‐way analysis of variance (ANOVA) followed by Dunnett's multiple‐comparisons test (when comparing more than two groups). In a few instances (comparison of only two groups) where data did not follow normal distribution, the non‐parametric two‐tailed Mann–Whitney U‐test was used. The Pearson correlation coefficient was used for correlation analyses. *p* < 0.05 were considered to be statistically significant.

## Author Contributions

Q.W. designed and performed research, analyzed and interpreted data, and wrote the manuscript. W.L. and Q.Y. analyzed and interpreted data, mainly scRNA and LC‐HRMS sequencing data. K.L. and H.W. interpreted data and contributed to writing. X.Z., S.J., D.Z., W.W., S.C., and Y.L, contributed to experiments. B.Y. interpreted data and edited the manuscript. Q.Y., Q.Y., and Y.W. conceived and designed the study, supervised research, interpreted data, and co‐wrote the manuscript.

## Conflict of Interest

The authors declare no conflict of interest.

## Supporting information



Supporting Information

Supplemental Table 1

Supplemental Table 2

## Data Availability

The LC‐HRMS data that support the findings of this study are openly available in the OMIX, Chinese Academy of Sciences at https://ngdc.cncb.ac.cn/omix: accession no. OMIX004846; The scRNA‐seq data of gingival mucosa have been deposited previously in the Gene Expression Omnibus (GEO) database, reference number GSE188217.
