# Supplementary material for: Hyperglycemia‐Enhanced Neutrophil Extracellular Traps Drive Mucosal Immunopathology at the Oral Barrier
Source: Adv Sci (Weinh). 2024 Nov 5;11(47):2407346. doi: 10.1002/advs.202407346 (PMC11653653; doi:10.1002/advs.202407346)
Supplement: Supplementary file 1 — Supporting Information [file ADVS-11-2407346-s001.pdf]

ADVANCED SCIENCE
Open Access

Supporting Information

for *Adv. Sci.*, DOI 10.1002/advs.202407346

Hyperglycemia-Enhanced Neutrophil Extracellular Traps Drive Mucosal Immunopathology at the Oral Barrier

Qian Wang, Weimin Lin, Kexin Lei, Hui Wang, Xiaohan Zhang, Shuang Jiang, Danting Zhang, Wen Wang, Shuqin Cao, Yuyu Li, Bo Yu, Yuan Wang, Qi Yin* and Quan Yuan**

Appendix for

Hyperglycemia-enhanced neutrophil extracellular traps drive mucosal immunopathology at the oral barrier

Qian Wang, Weimin Lin, Kexin Lei, Hui Wang, Xiaohan Zhang, Shuang Jiang, Danting Zhang, Wen Wang, Shuqin Cao, Yuyu Li, Bo Yu, Yuan Wang*, Qi Yin *, Quan Yuan *

*Correspondence:

yuanquan@scu.edu.cn; yq@scu.edu.cn; wangyuan2117@scu.edu.cn

Running title: Hyperglycemia-induced NETs drive gingival immunopathology

Key words: Hyperglycemia; Oral mucosal immunity; Neutrophils; Type 2 diabetes; Glycolysis; Glucose transporter 1

This file includes:

1. Supplementary Figures and Legends
2. **qPCR** primer

Supplementary Figure

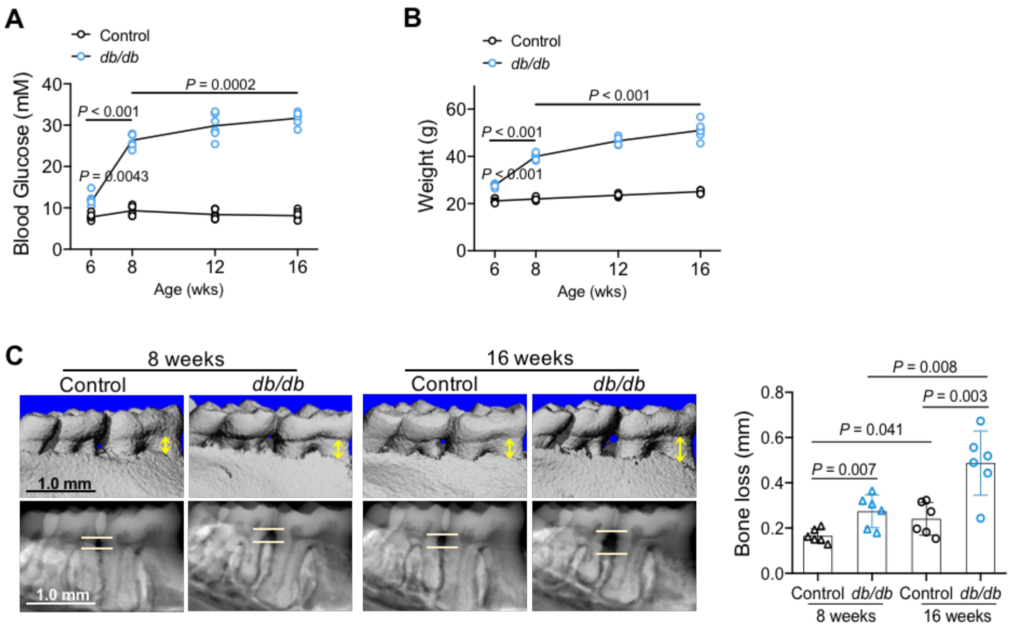

Figure S1. Establishment of T2D mouse model and periodontal bone loss in *db/db* mice. (**A**) Blood glucose levels after 6 h fasting immediately from 6- to 16-week-old in control and *db/db* mice. (**B**) Detection of weight in control and *db/db* mice. Results are shown as mean ± SD, n = 6, Student's *t* test. (**C**) Micro-CT visualization of periodontal damage in 8- and 16-week-old *db/db* mandibles. (Top) Three-dimensional reconstructed images; (bottom) X-ray observation. Scale bar: 1 mm. Yellow arrows and lines indicate the distance between the alveolar bone crest and the cementoenamel junction (ABC-CEJ). Right: measurements of periodontal bone loss for *db/db* and control littermates, n = 6.

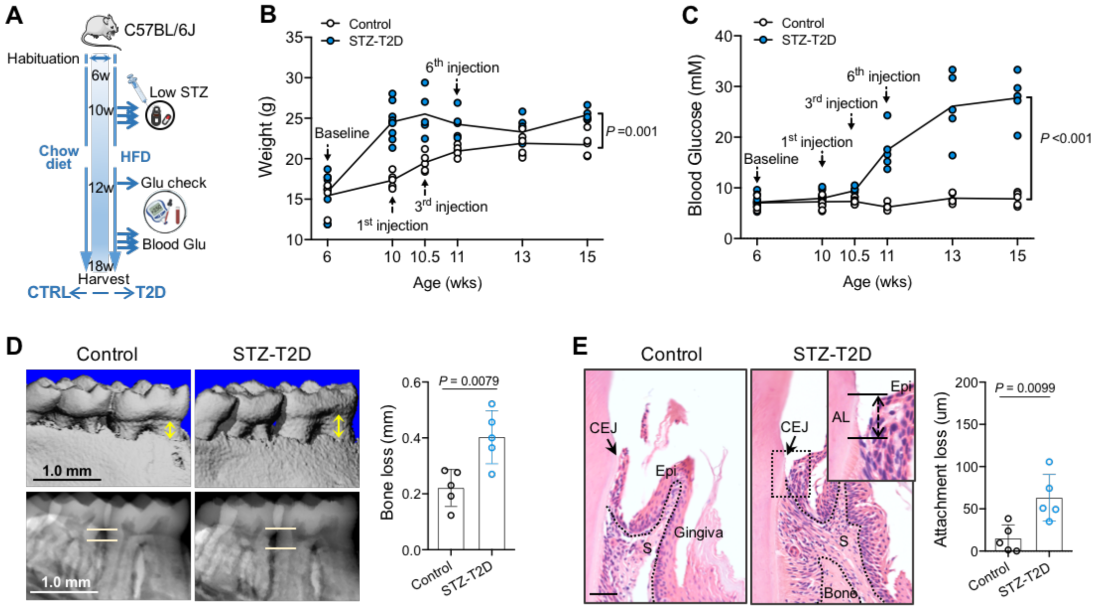

Figure S2. Combination of HFD and low-dose STZ induces T2D in mice.

(**A**) Schematic illustration of a high-fat diet (HFD)/streptozotocin (STZ)-induced T2D mouse model (STZ-T2D). (**B** and **C**) Detection of weight and blood glucose levels in control and STZ-T2D mice, beginning at one day before STZ injection. Results are shown as mean ± SD, n >= 5, Student's *t* test. (**D**) Micro-CT visualization and quantification of periodontal bone loss in STZ-T2D mandibles (yellow arrows and lines indicate the distance between the ABC-CEJ), n = 5. Scale bar: 1 mm. (**E**) H&E staining of STZ-T2D oral mucosal tissue sections (black arrowhead depicts CEJ and black lines indicate mucosal attachment loss). Right: quantification of the attachment loss. AL, attachment loss; CEJ, cementoenamel junction; Epi, epithelium; S, stroma. Scale bar: 50 µm.

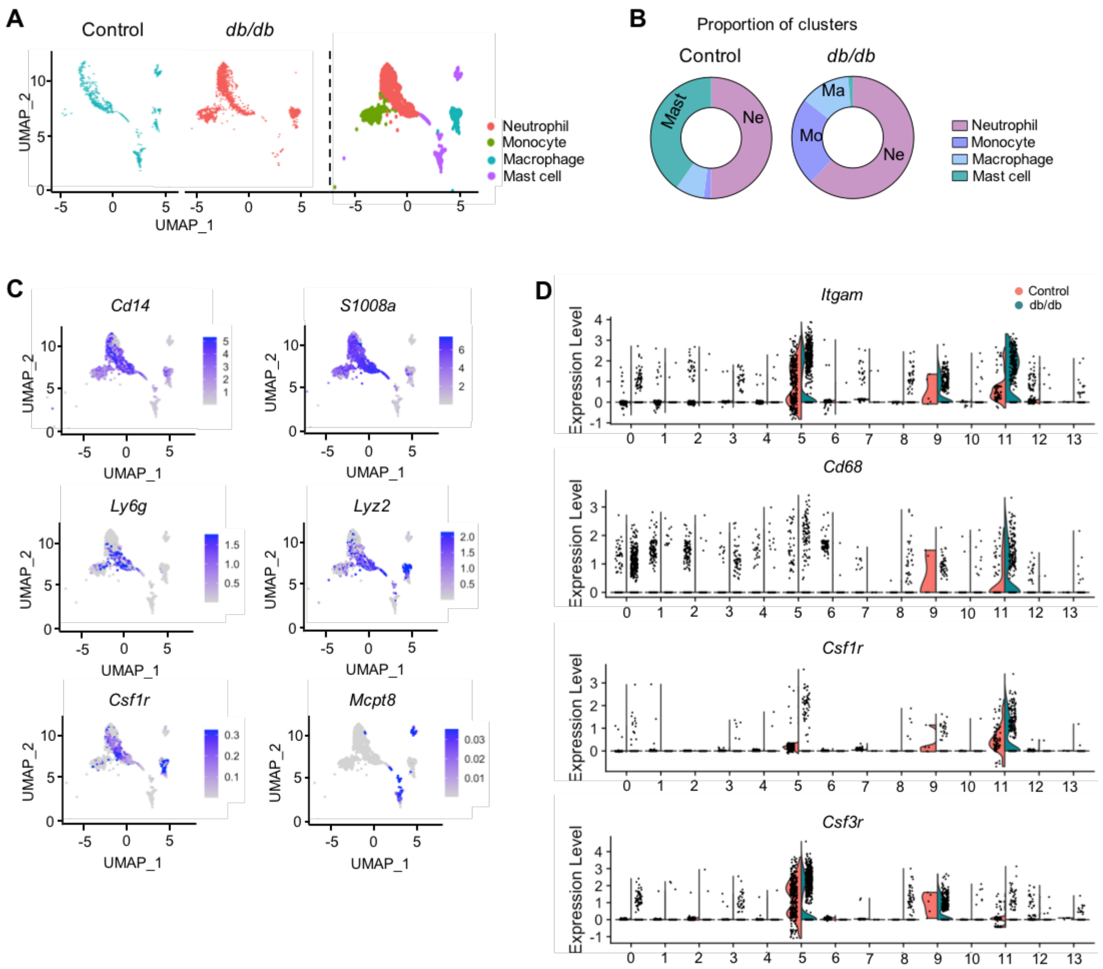

Figure S3. Identification of myeloid subsets in gingival mucosal atlas.

(**A**) Two-dimensional UMAP representation of myeloid **subsets**, according to (left) sample origin and (right) results of clustering. (**B**) Proportion plot of cell sub-clusters in myeloid cells. (**C**) Expression levels of marker genes of myeloid cells (neutrophil, monocyte, macrophage and mast cell) were projected onto UMAP atlas, including *Cd14*, *S1008a*, *Ly6g*, *Lyz2*, *Csf1r* and *Mcpt8*. (**D**) Violin plots visualization of the differential expressed genes in 14 clusters, which are involved in myeloid cells development between two groups' gingival tissues.

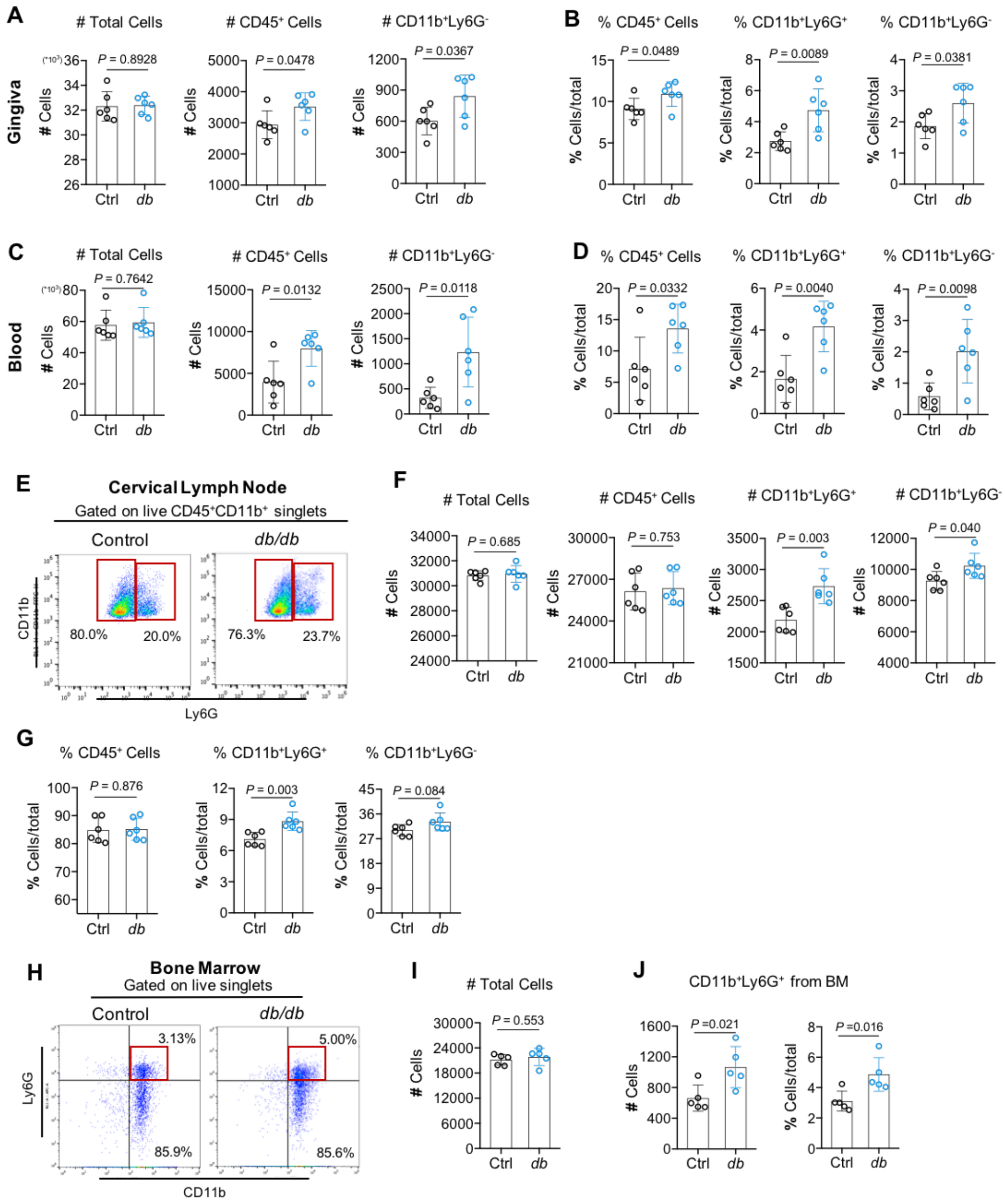

Figure S4. Increased neutrophil mobilization in *db/db* mice.

(**A-B**) Representative FACS plots for identification of neutrophils in16-week-old mouse gingival tissues. After gating for CD45$^+$ cells, neutrophils are identified as CD11b$^+$Ly6G$^+$ cells. CD45$^+$ populations are further characterized as other myeloid cells (CD45$^+$CD11b$^+$Ly6G$^-$). Absolute cell numbers (A) of total, CD45$^+$ and CD11b$^+$Ly6G$^-$ cells; and frequencies (B) of the same populations in total gingival cells. (**C-D**) Representative FACS plots for identification of neutrophils from 16-week-old mouse blood. Absolute cell numbers (A) of total, CD45$^+$ and CD11b$^+$Ly6G$^-$ cells; and frequencies (B) of the same populations in total blood cells. (**E-G**)

Flow cytometry analysis of mouse cervical lymph nodes proximity to gingival immunopathology tissues detected above. Representative FACS plots (E) to identify neutrophils ($CD45^+CD11b^+Ly6G^+$) and $CD45^+CD11b^+Ly6G^-$ populations, absolute counts (F) and frequency (G) of the same populations in total cells of cervical lymph nodes. (**H**) Representative FACS plots to identify $CD11b^+Ly6G^+$ populations in bone marrow (BM) from the same mouse detected above. (**I**) Graphs showing absolute count of total cells obtained from control and *db/db* bone marrow. (**J**) (left) Absolute cell numbers of neutrophil ($CD11b^+Ly6G^+$) and (right) frequency in BM.

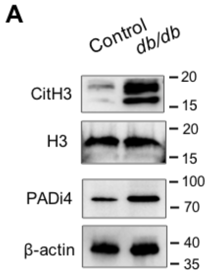

Figure S5. Increased levels of NETs in gingival tissues of *db/db* mice.

(**A**) Western blot analyses of PADi4 enzyme and citrullinated core histones H3 (CitH3) in control and *db/db* gingival mucosal tissues.

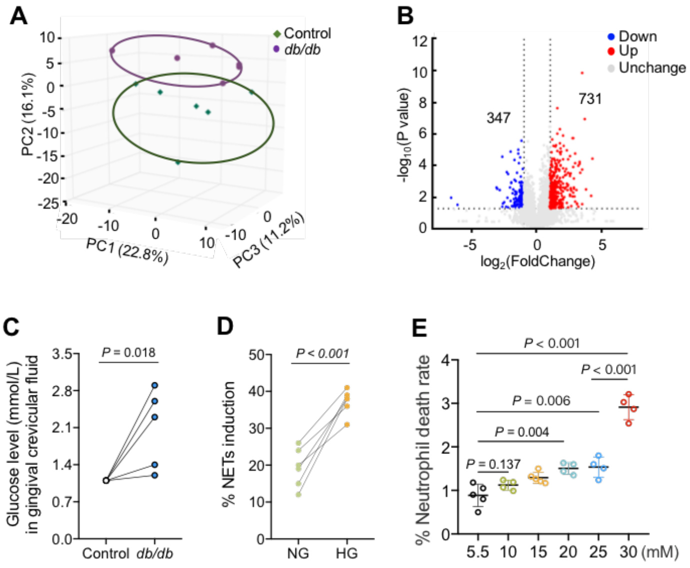

Figure S6. High Glucose induces neutrophil extracellular traps in T2D mice.

(**A**) Principal component analysis (PCA) plot in positive mode showing the distribution of differential metabolites in oral mucosa of control and *db/db* mice. (**B**) Volcano map displaying the differential metabolites in *db/db* gingival tissues compared to that of control group, with 731 upregulated metabolites and 347 downregulated metabolites. (**C**) Detection of glucose levels about gingival crevicular fluid in control and *db/db* mice. n=5. (**D**) Quantification of the percentage of NET induction (co-localized CitH3 and DAPI) for Fig. 4H. (**E**) Quantification of percentage of bone marrow derived-neutrophils death in different glucose concentrations.

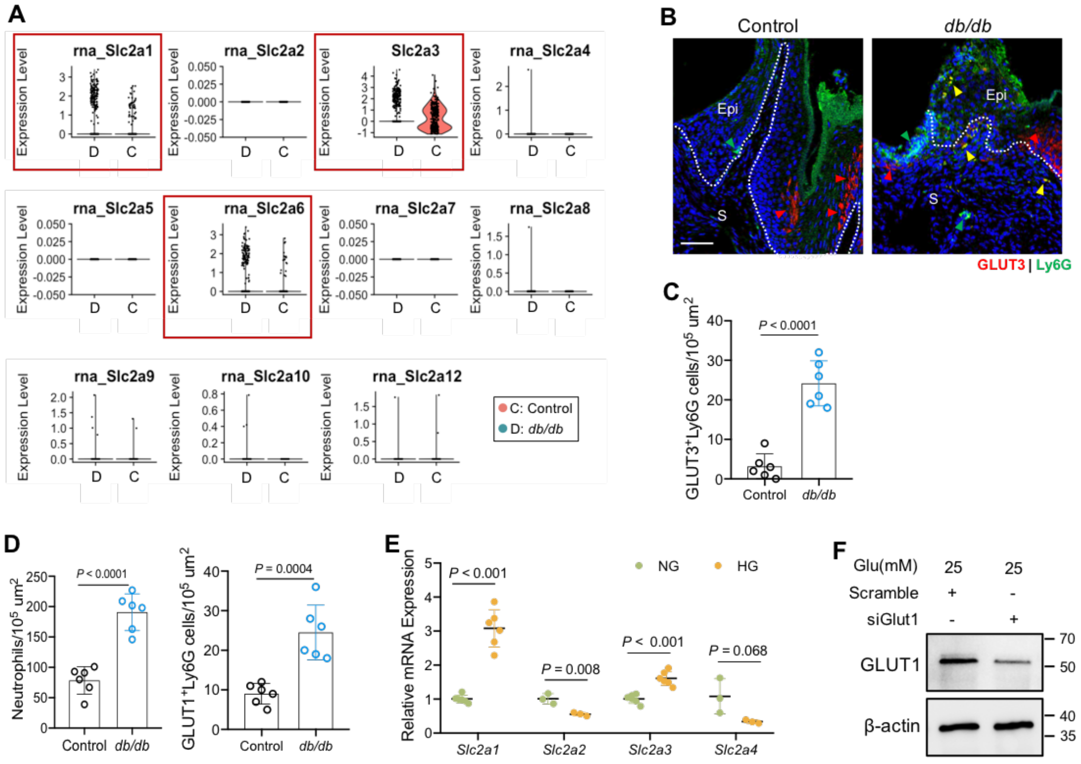

Figure S7. Neutrophil NETosis is dependent of elevated GLUT1 expression.

(**A**) Violin plots showing solute carrier family (SLC) mRNA expression level in neutrophil sub-cluster between control and *db/db* mice. (**B**) IF staining of GLUT3 (red) and Ly6G (green) in *db/db* oral mucosal tissue sections. Yellow triangles indicate the co-localized cells. Scale bar, 50 μm. Epi, epithelium; S, stroma. (**C**) Quantification of the number of GLUT3^{+}Ly6G^{+} cells per 10^5 μm^2 of stained tissue sections. (**D**) Quantification of the number of neutrophils and GLUT1^{+}Ly6G^{+}- cells per 10^5 μm^2 of stained tissue sections. (**E**) Expressions of different glucose transporters (*Slc2a1-4*) mRNA are measured by *qPCR*, normalized to *Gapdh*. BM neutrophils from control mice are stimulated with 5.5 mM and 25 mM glucose for 3 h. (**F**) siRNA treatment. Expression of GLUT1 protein is evaluated by western blot.

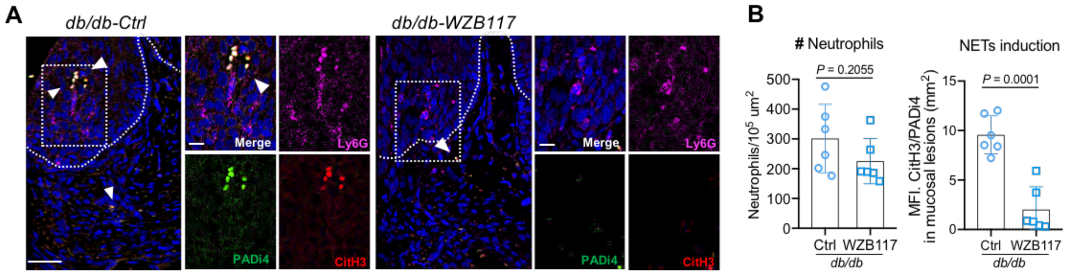

Figure S8. Inhibition of GLUT1 reduces NETs formation.

(**A**) IF staining of Ly6G (purple), CitH3 (red) and PADi4 (green) in gingival tissue from *db/db* mice with or without WZB117 treatment, where NETs are indicated by white triangles. Scale bars: low magnification, 30 µm; high magnification, 10µm. (**B**) Percentage of Ly6G, CitH3 and PADi4 stained area, respectively. Left: graphs showing the number of neutrophils ($Ly6G^+$) per 10^5 μm^2 of stained tissue sections. Right: NETs are quantified by the MFI of CitH3 or PADi4 staining in oral mucosal lesions, and the ratio of CitH3 to PADi4 is calculated.

The *qPCR* primer used in this study

Genes	Forward（5’-3’）	Reverse（5’-3’）
Slc2a1	GAGACCAAAGCGTGGTGAGT	GAGTTCGGCTATAACACTGG
Slc2a2	ATCCCTTGGTTCATGGTTGCTG	TCCGCAATGTACTGGAAGCAG
Slc2a3	ATCGTGGCATAGATCGGTTC	TCTCAGCAGCTCTCTGGGAT
Slc2a4	CCAGCCACGTTGCATTGTA	ACACTGGTCCTAGCTGTATTCT
Gapdh	ACCCTTTCACTGGGGATCACA	GACAGGGATCAGGATTTCCTTG
Human		
IL24	CTTTGTTCTCATCGTGTCACAAC	TCCAACTGTTTGAATGCTCTCC
AHR	CAAATCCTTCTAAGCGACACAG	TGACG-CTGAGCCTAAGAACA
GAPDH	CCTGTTCGACAGTCAGCCG	CGACCAAATCCGTTGACTCC
